# Supplementary figures and images for: Seeking safety: Movement dynamics after post-contact immobility
Source: PLoS One. 2024 Aug 22;19(8):e0307370. doi: 10.1371/journal.pone.0307370 (PMC11340899; doi:10.1371/journal.pone.0307370)

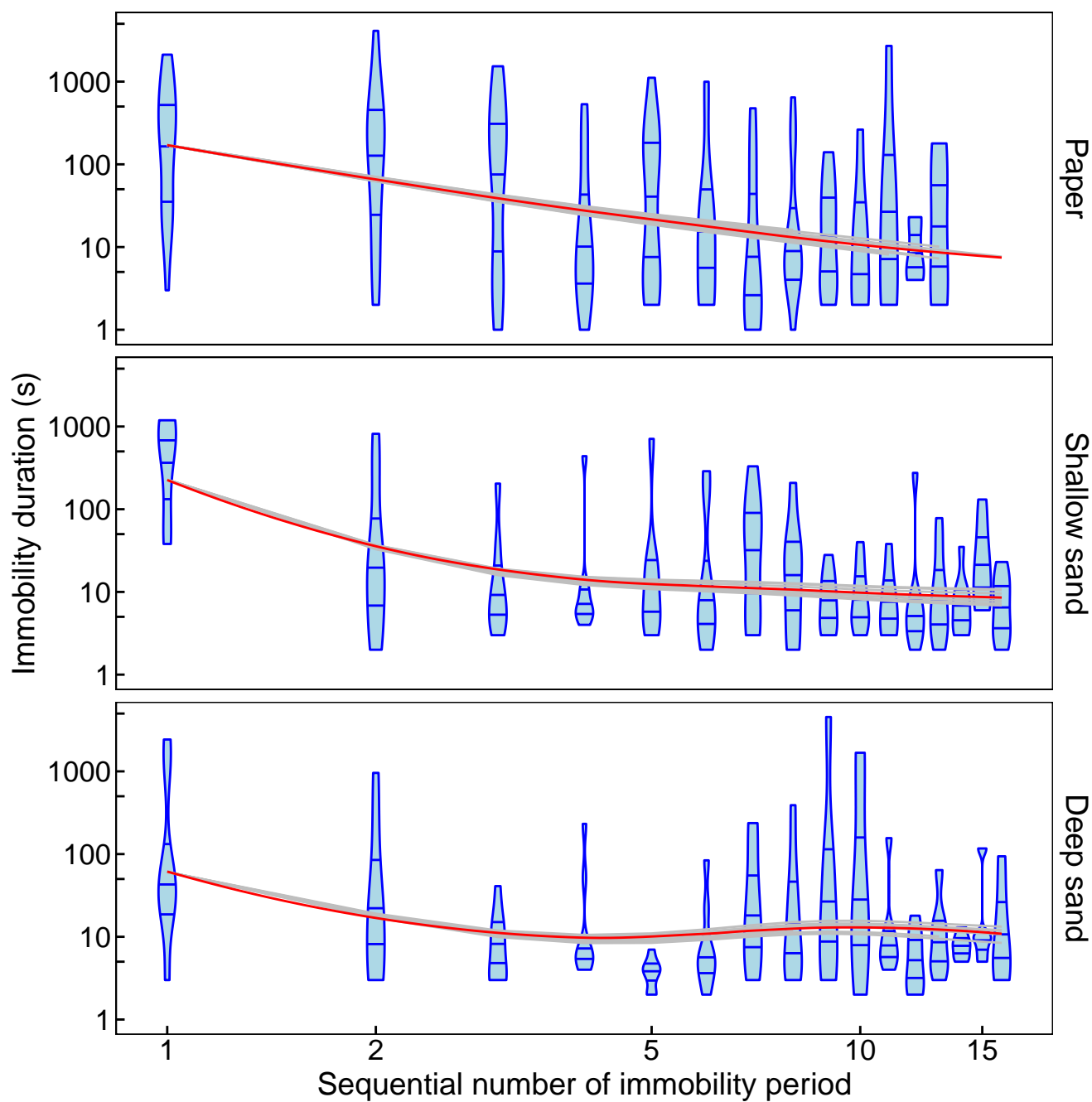

Supplement: S1 Fig — It is the same as Fig 1 except that the fitted lines are not based on the assumption of a straight-line relationship but are smoothers from a GAMM instead; both axes are on a log scale; blue “violins”: mirror density plots with horizontal lines representing the median, upper and lower quartile, red line: a smoother for the overall relationship (predicted fixed effects from the model), grey line: a smoother for each individual antlion (predicted random effects from the model). (PDF) [file pone.0307370.s002.pdf]

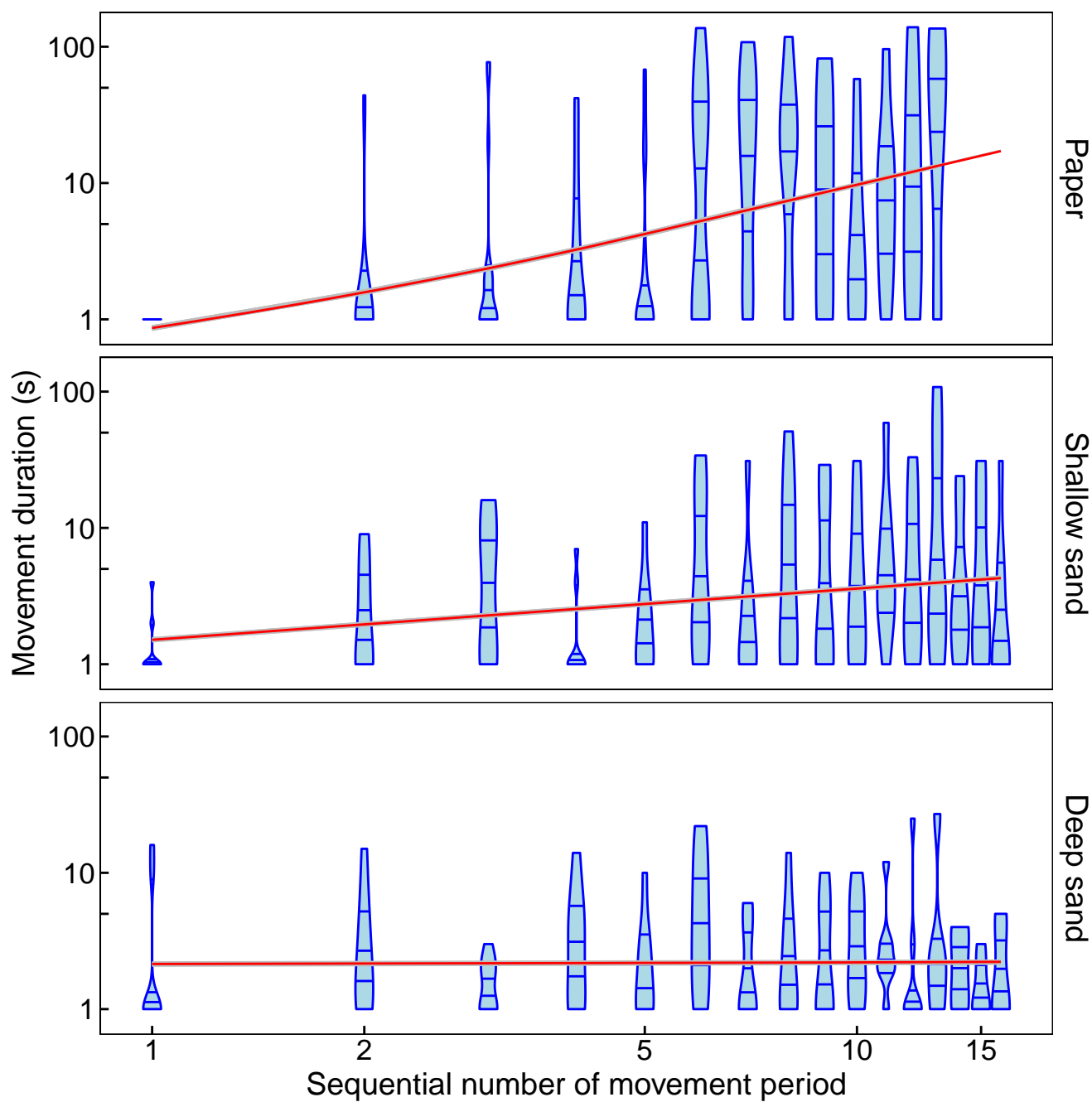

Supplement: S2 Fig — It is the same as Fig 2 except that the fitted lines are not based on the assumption of a straight-line relationship but are smoothers from a GAMM instead; both axes are on a log scale; blue “violins”: mirror density plots with horizontal lines representing the median, upper and lower quartile, red line: a smoother for the overall relationship (predicted fixed effects from the model), grey line: a smoother for each individual antlion (predicted random effects from the model, very similar within each treatment here). (PDF) [file pone.0307370.s003.pdf]

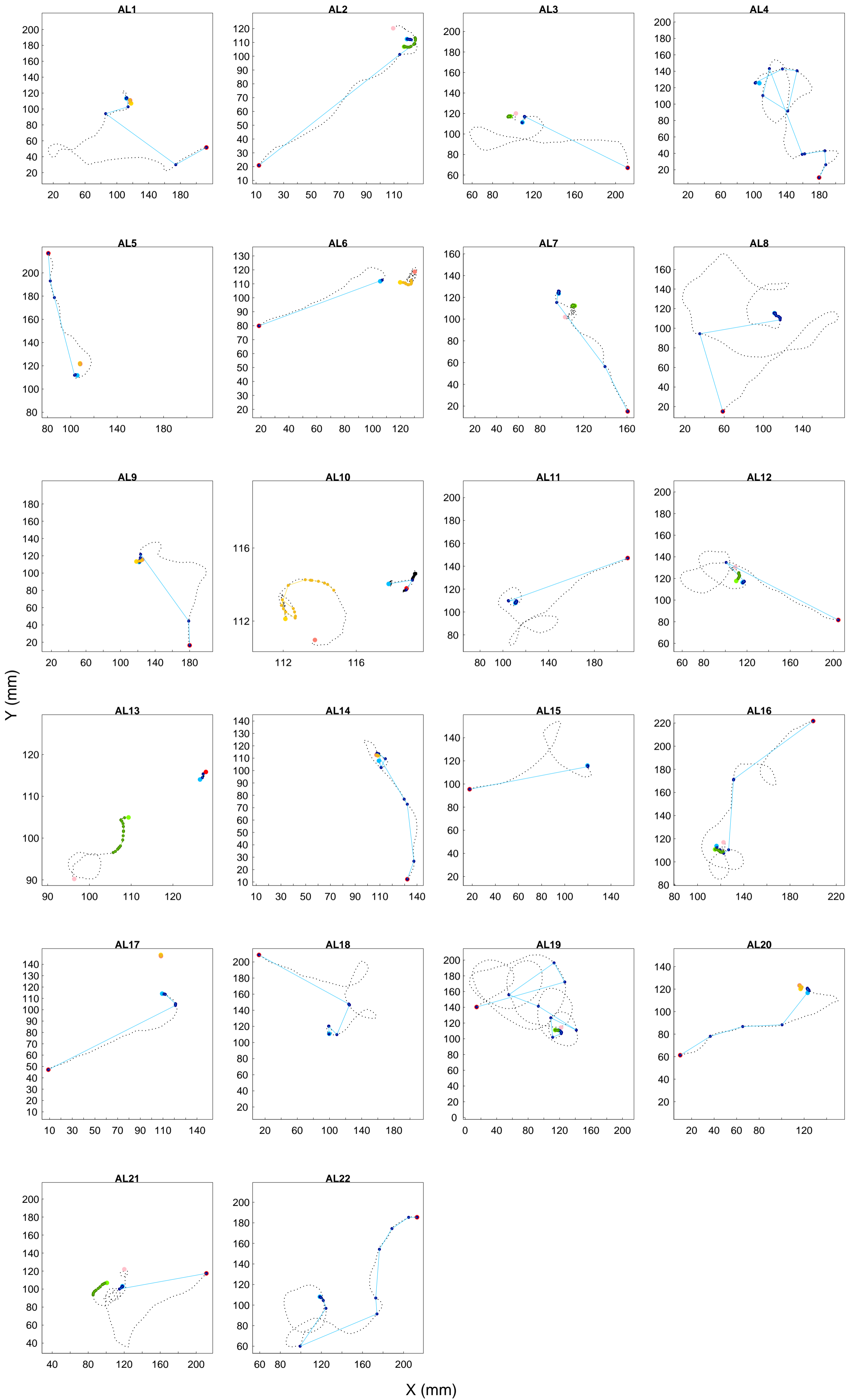

Supplement: S3 Fig — (PDF) [file pone.0307370.s004.pdf]

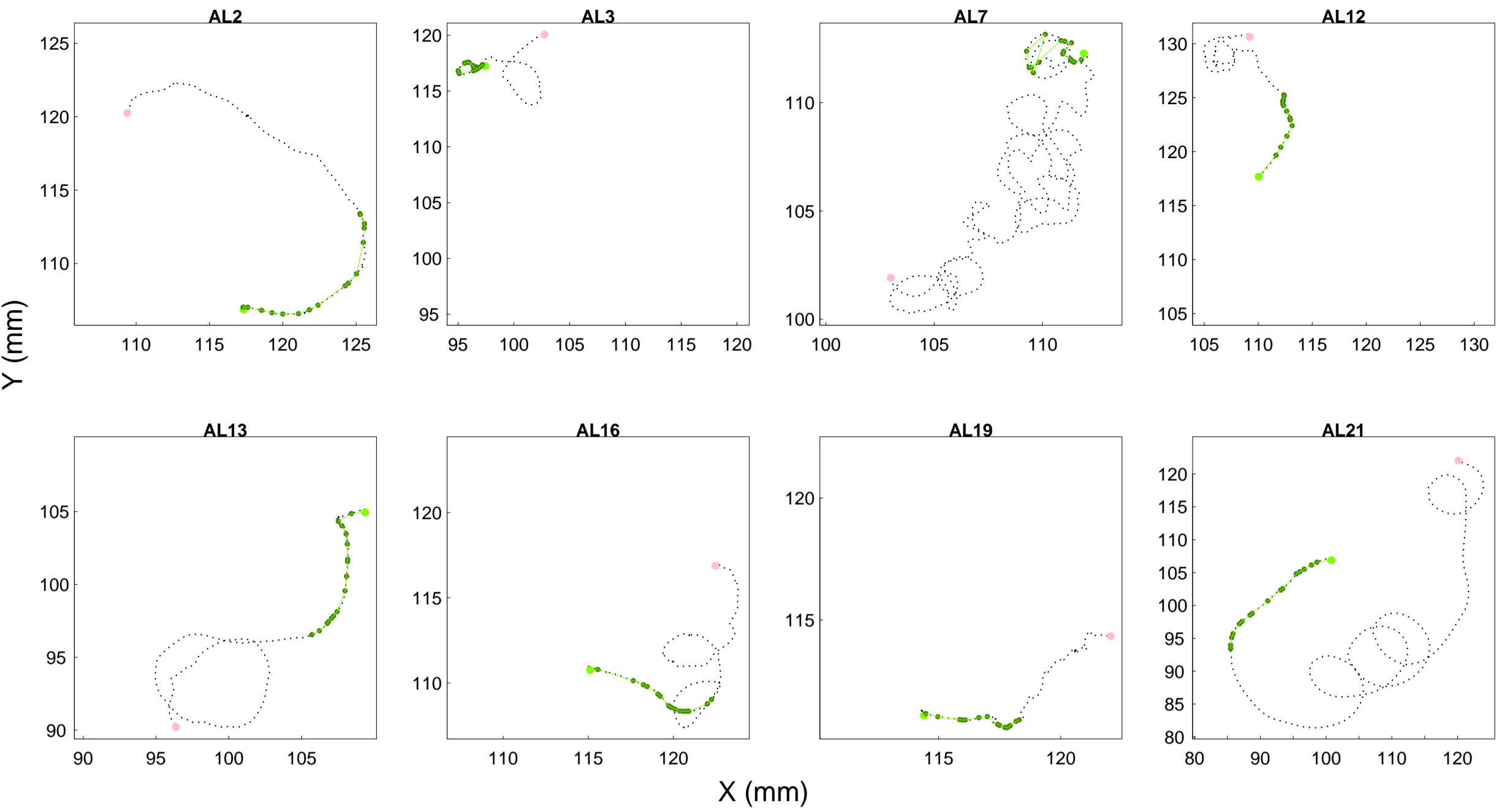

Supplement: S4 Fig — (PDF) [file pone.0307370.s005.pdf]

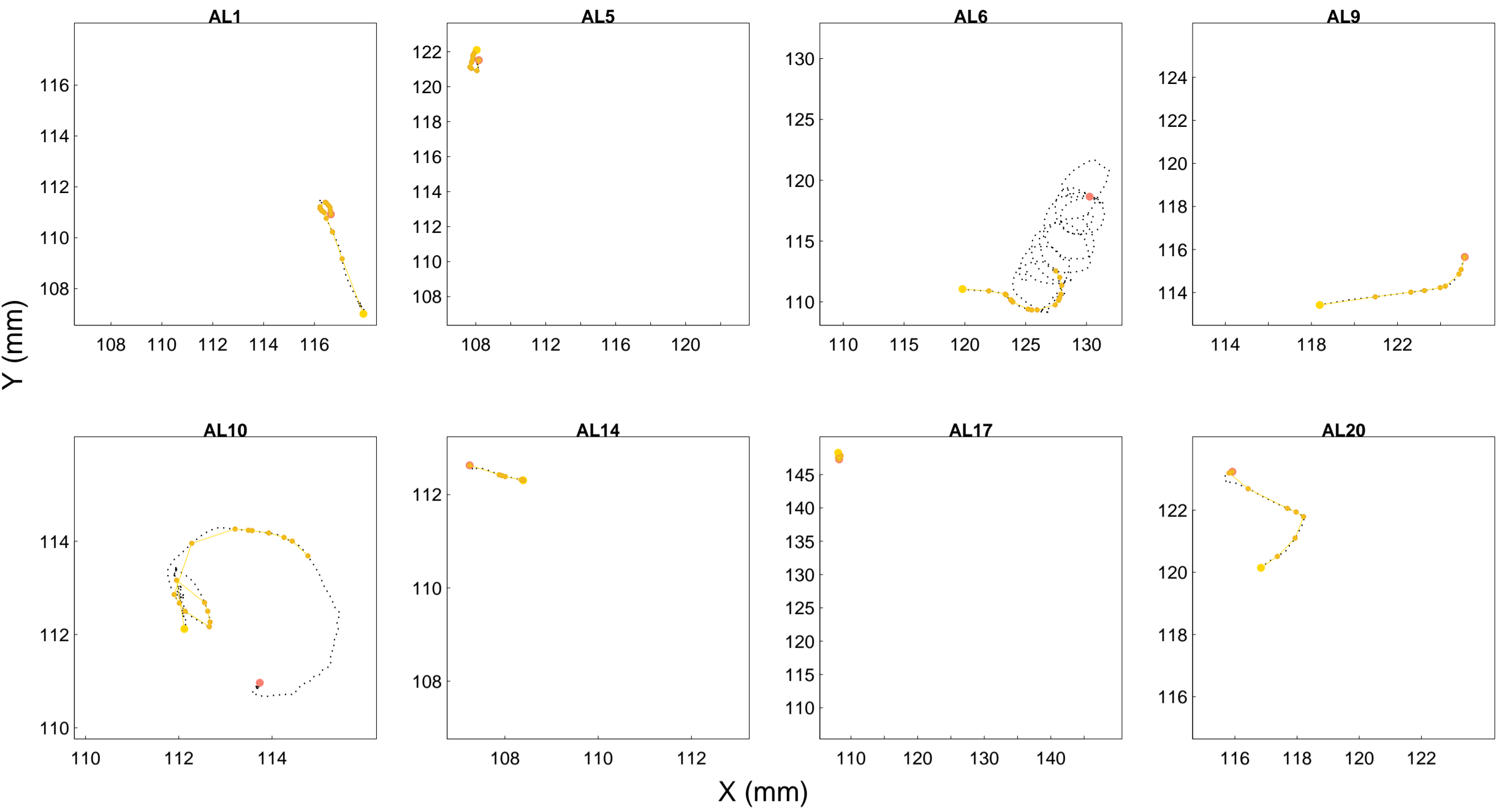

Supplement: S5 Fig — (PDF) [file pone.0307370.s006.pdf]

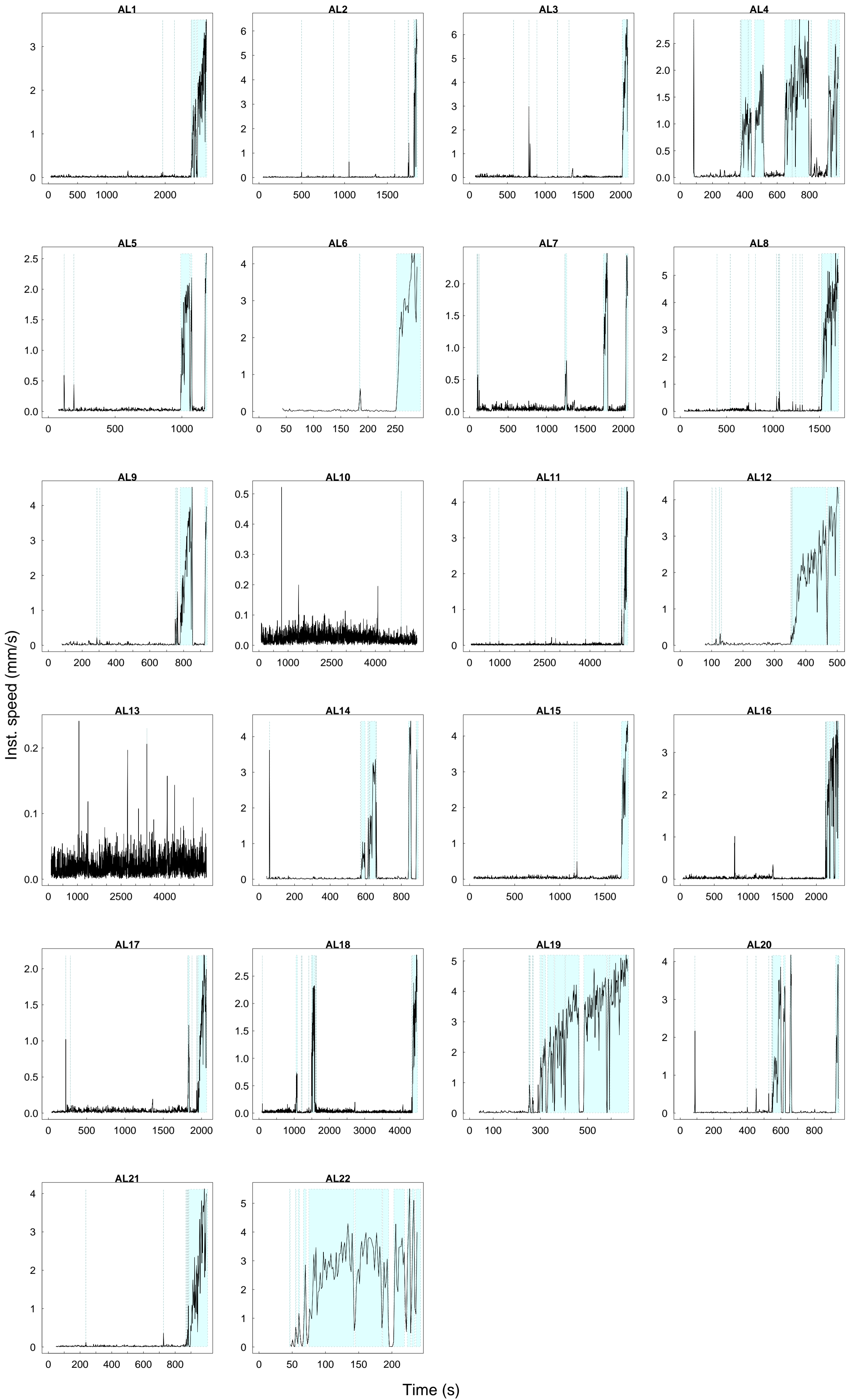

Supplement: S6 Fig — (PDF) [file pone.0307370.s007.pdf]

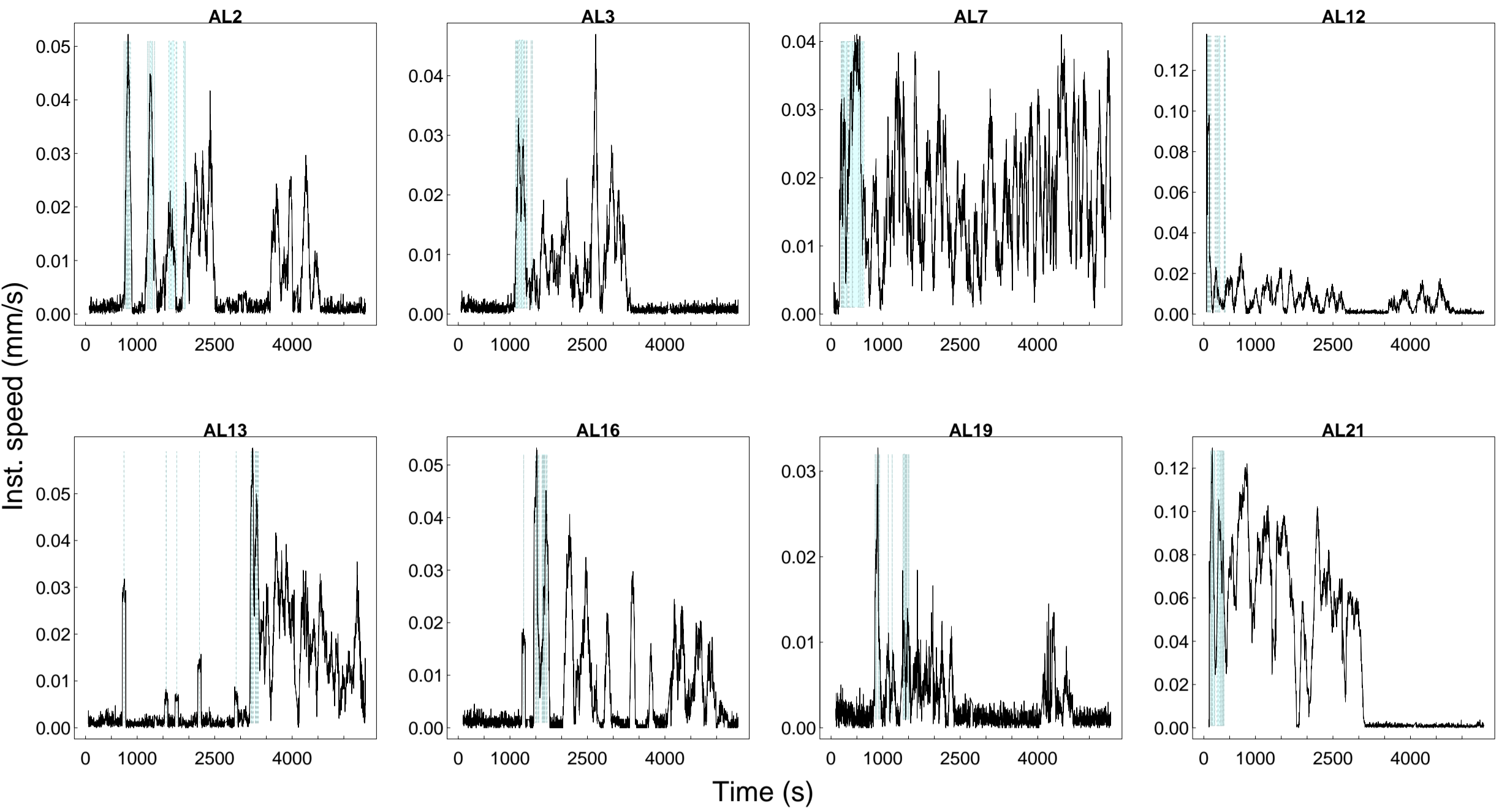

Supplement: S7 Fig — (PDF) [file pone.0307370.s008.pdf]

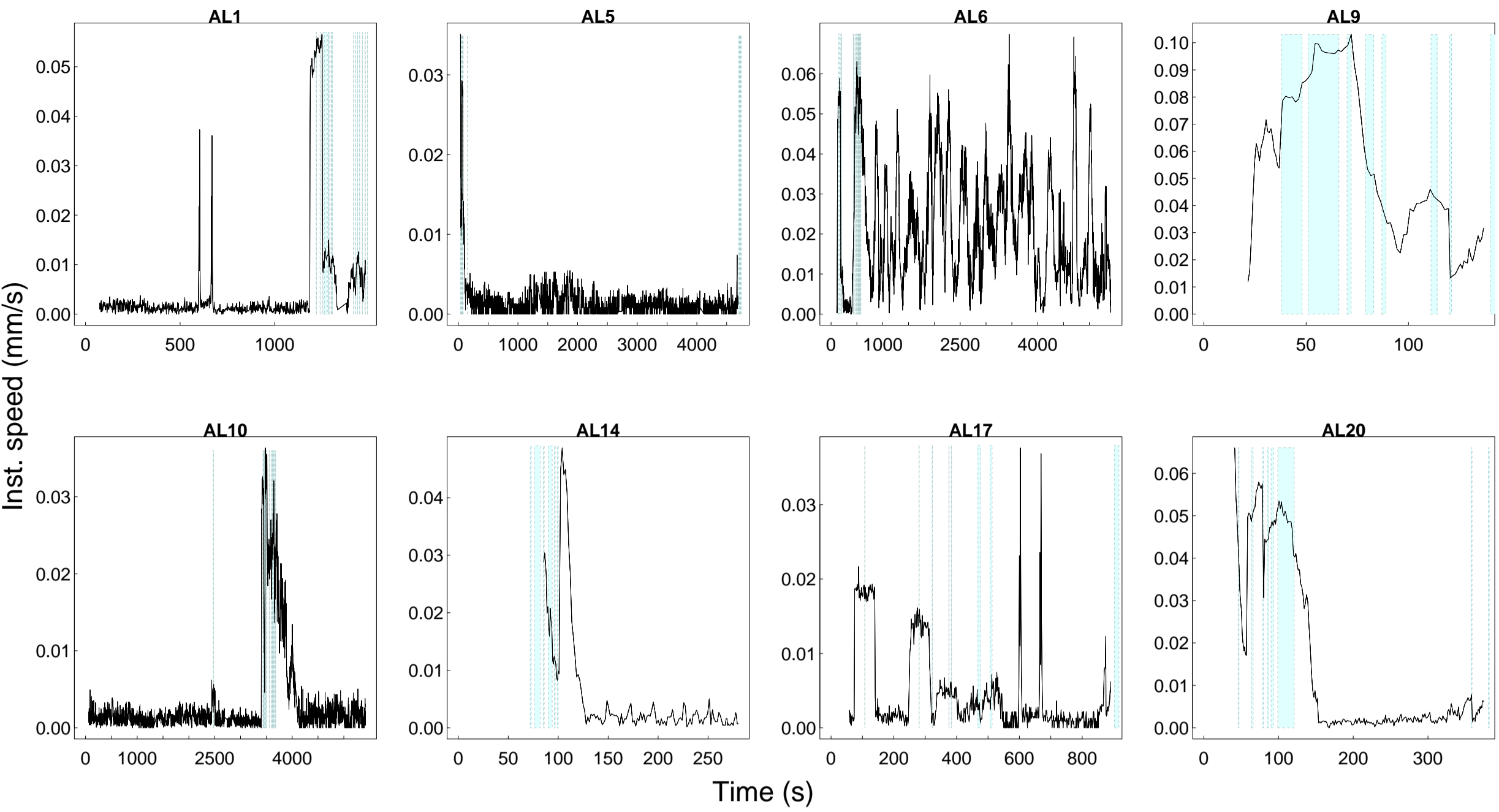

Supplement: S8 Fig — (PDF) [file pone.0307370.s009.pdf]
