# Supplementary material for: Seeking safety: Movement dynamics after post-contact immobility
Source: PLoS One. 2024 Aug 22;19(8):e0307370. doi: 10.1371/journal.pone.0307370 (PMC11340899; doi:10.1371/journal.pone.0307370)
Supplement: Attachment — Submitted filename: Response to Reviewers PONE-D-23-29142.docx [file pone.0307370.s016.docx]

**PONE-D-23-29142**

**Seeking safety: movement dynamics after post-contact immobility**

1. Reviewer #1: Dear authors, I have read your study entitled »Seeking saftey: movement dynamics after post-contact immobility«. It is an interesting study with general appeal, and knowing your previous studies, it provides additional information on how antlions respond to a putative predator after post-contact immobility.

**Thank you.**

2. Your statistical methods are quite extensive. You have taken a very quantitative approach to your study. I think this is good, but to balance this we also need a simple description of behaviour that best illustrates the actual state of the animal under and after PCI. Measuring behaviour using mostly a statistical approach is not enough. This is only consideration. The description of behaviour and, in addition, physiological measurements can provide important information about whether the animals are under stress or not. The biological background of these animals is also important. E. nostras is a habitat specialist that lives in sheltered places and avoids direct sunlight.

**Thank you for these considerations. We have described the behaviour of PCI in a previous study (Sendova-Franks et al.. 2020, [9]) and it is typical of other descriptions of this phenomenon, which we also cite extensively. We agree that the physiological approach is important but we are not physiologists and hope that our study will encourage physiologists to measure the physiological characteristics of PCI in insects. We agree that mapping the physiological characteristics to the behaviour quantification we have used would further our understanding of this phenomenon within and across species (LL. 104-106, 602-603, 630-632).**

3. Some conditions in your study may have affected your measurements, especially given the small number of animals studied.

**Crucially, such conditions are not conflated with the variables we are trying to measure. Please see our responses to specific comments below.**

4. In some parts of the discussion you make comparisons with other studies, which I think is hardly possible based on the results of your study. In general I like your study, but biological background conected to animals you tested is somewhat missing.

**We are very glad Reviewer #1 likes our study and have addressed the specific comments below.**

5. I have some minor comments on some parts of the manuscript that I think need to be explained:

Keywords: you use different keywords for the same thing. You have particularly focused on mobility after PCI.

I have some minor comments on some part of the masucript which I think needs to be explained:

Key words: you use different key words for the same thing. You focused especially on mobility after post-contact. Mybe you consider deleting some and add »antlion larvae«, »sit-and-wait animals« as this could be important for other studies that you elucidate you had sit-and-wait animals.

**Depending on whether we could make changes to the keywords, we will endeavour to include “antlion larvae” and “sit-and-wait predator” (LL. 49-50).**

6. Line 57: mybe you add (in our case antlions)

**We would like to keep things as they are so that we begin with the main issues we aim to address and only then introduce our model system (in the third paragraph of the Introduction).**

7. Line 79: »can span more than three orders of magnitude« why not just write »can last«

**We think the point about the three orders of magnitude is very important and the correct verb for it is span not last.**

8. Lines 82-86: you explain some statistical data acording to previous studies. They are not necessary. Just continue with line 87.

**We would like to keep these lines because they give evidence for the statement that our findings are consistent with earlier work and the reason why PCI duration is unpredictable.**

9. Line 91: delete »an endless future«

**We would like to keep this expression.**

10. Line 125: mybe you should add »sit-and-wait organisms« as this could be important in comparison of PCI and movements after that in comparison to other more active animals.

**Done (L. 125).**

11. Line 131: it is not an option it is innate fixed-action pattern when antion are on surface. Consider rewriting sentence.

**We would like to keep ‘an option’ here because we are making a general point about the next step to safety for antlion larvae coming out of PCI on any surface. When this surface is impenetrable, they cannot dig. Considering digging as a fixed-action pattern is inappropriate since if that was the case an antlion larva that has come out of PCI on an impenetrable surface would stay on the same spot trying to dig a pit rather than move away.**

12. Line 136: is 4 mm really deep sand? You refer further that 2 and 4 cm was used in previous studies, which is definetely more appropriate for deep and shallow sand. And as you explain further in such sand the pit-building is not possible. True, but also submergence in 4 mm sand is not quite possible for the third instar (larger larvae). The larva hits the hard surface beneth before it is fully submerged.

**It is true that while the majority of 3^rd^ instar larvae would be able to submerge themselves completely in 4.6 mm-deep sand (L. 199-201), the largest possible 3^rd^ instar larvae might just have the tip of their thorax sticking out (LL. 562-563). Still their submergence would be much more successful in Deep sand than in Shallow sand.**

13. Line: what is fine-tuned ability? Please explain.

**We have amended this to ‘evolutionarily fine-tuned’ (L. 155).**

14. Line 162: what do you mean »two sand substrates«? You mean two sand depth? If you refer to »two subsrates« it can be understand you had different coarsesnes.

**Thank you. We have changed this to ‘two sand treatments’ because difference in sand depth is mentioned in the following sentence (L. 164).**

15. Line 161-164: it is not very clear what you are trying to formulate. The larvae have innate behaviour to burry under the substrate after they find themself on the surface. Untill they start burrying themself they can not percive the difference in sand depth – so this question is not understandable. Moreover, you had different larval stages and they probably experienced sand depth in different ways.

**As we state earlier on in the Introduction (LL. 126-128), Farji-Brener et al. (2022) Animal Behaviour ([10]) demonstrated that antlion larvae have different PCI durations on different substrates (compact vs loose soil). This shows that they do not have to start burying themselves before they can perceive the characteristics of the substrate. We did not find any effect of larval weight in our models (LL. 304-306).**

**Please note that comments #16 to #26 below are copies of comments #5 to #15 above, probably due to an error with Copy and Paste. Please refer to the responses under comments #5 to #15 above.**

16. . Maybe you should delete some and add »antlion larvae«, »sit-and-wait animals« as this might be important for other studies

**Please see response to comment #5 above.**

17. Line 57: maybe add (in our case antlions)

**Please see response to comment #6 above.**

18. Line 79: »can span more than three orders of magnitude«, why not just write »can take/last« or something similar

**Please see response to comment #7 above.**

19. Lines 82-86: you explain some statistical data based on previous studies. They are not necessary. Just continue with line 87.

**Please see response to comment #8 above.**

20. Line 91: delete »an endless future«

**Please see response to comment #9 above.**

21. Line 125: perhaps you should add »sedentary organisms« as this might be important for comparing PCI and subsequent movements compared to other, more active animals.

**Please see response to comment #10 above.**

22. Line 131: it is not an option but an innate behavioural pattern when the antions are on the surface. Perhaps you should rewrite the sentence.

**Please see response to comment #11 above.**

23. Line 136: is 4 mm really deep sand? You point out that previous studies have used 2 and 4 cm, which is definitely more appropriate for deep and shallow sand. Is complete submersion in 4 mm deep sand possible for the third instar (larger larvae)? The larva hits the hard surface before it is fully submerged.

**Please see response to comment #12 above.**

24. Line: What is a fine-tuned ability? Please explain.

**Please see response to comment #13 above.**

25. Line 162: What do you mean by »two substrates of sand«? Do you mean two depths of sand? If you are referring to »two substrates«, it can be assumed that you had different coarsenesses.

**Please see response to comment #14 above.**

26. Lines 161-164: It is not entirely clear what you are trying to formulate. The larvae have an innate behaviour to bury themselves under the substrate after they are on the surface. As long as they do not bury themselves, they cannot perceive the difference in sand depth – so this question is not understandable. Also, you had different larval stages that probably perceived the sand depth in different ways.

**Please see response to comment #15 above.**

27. Line 170: »most larval stages« define what larval stages you had. With the measurements of the head and body this is easy, but according to your mass you probably had the 2nd and 3rd instar. It might be difficult to manipulate with the first instar – also, it's probably difficult to compare the duration of movements in such different sized animals. Probably the first or second instar needs greater challenges to move through the substrate and walks less and stops more often. It is very speculative to observe and define behaviour in such a small number of test animals and such a variety of stages. Just one consideration.

**We have edited this sentence to read “Larval weight covered a wide range and likely represented the 2^nd^ and 3^rd^ larval stages (median = 0.0360 g, LQ = 0.0183 g, UQ = 0.0850 g, S1 Table) (LL. 173-175)”. We used both video recording and image analysis to analyse the movements of all individuals. We found no effect of antlion weight in our models in both the present and earlier studies of movement and immobility (LL. 304-306). Our sample sizes have statistical power and our significant results demonstrate that the biological signal is strong. Antlions are rare on the British Isles. We sampled a small population on Guernsey in an as unbiased way as possible and think that our sample is representative. Please, see also response to comment #47 below.**

28. Line 173: Do you provide water to the larvae othervise? And how? What temperature were the larvae at when they were isolated? This could be important as temperature could significantly affect the duration of PCI as antlions are ectothermic organisms. Were the larvaein vials with sand or without sand when they were isolated? Please provide information.

**We have added the requested information. We did not provide food or water during the experiments either (L. 178). When the larvae were not taking part in experiments they were at room temperature (LL. 177-178). The room temperature range represented variation during the day (L. 176) and each substrate was tested in two session (morning and midday or early afternoon, LL. 184-185) to be representative of this range. When not taking part in the experiments, each larva was housed in its own vial without sand (L. 172).**

29. Line 174: perhaps it is more important that E. nostras is a habitat specialist that chooses places protected from rain and direct sunlight.

**We have added “…sit-and-wait predators that build their pits in places protected from rain and direct sunlight, spend most of their time in darkness at the bottom of their pits …” (LL. 178-180).**

30. Lines 184-186: you stated that they spend most of their lives in darkness, but in the experiment you placed lights over the arenas. Do you think this was not disruptive? And could it have an influence on the PCI and the behaviour of the animals after the PCI?

**The whole experiment simulated a disruptive event, namely a predator attack and subsequent dropping of the prey on the substrate. The same type of lighting was applied to all three substrate treatments so that the effect of substrate cannot be conflated with any effect of lighting.**

31. Line 187: You should specify what type of substrate particles you used for the study. Since you had larvae of different sizes, this is very important.

**We have added the following information: “decorative white sand, Trustleaf Aquasand, Natural White Silica Sand, pH Neutral, grain diameter between 0.250 and 0.500 mm …” (LL. 196-197).**

32. Line 228: You wrote that you observed the antlions for 90 minutes. Why did you stop after the antlion had reached the arena wall? Or have I misunderstood? It would be interesting to know how the antlions moved after hitting the arena wall. Did they return to the centre of the petri dish or did they just move around the edge. This is an important anti-predator behaviour that should be observed, especially in cases where burying is not possible.

**Antlions reached the wall only in the Paper (Hard substrate) treatment. Those that reached the wall in less than 90 min either moved a little along the wall or to the nearest corner and then stopped. They did not venture back into the centre of the experimental arena. We have added a sentence with this information (LL. 238-240).**

33. Line 264: Rather use "toss" or "throw" instead of "kick"

**We replaced “kicking up sand” with “throwing sand” (L. 274).**

34. Kind regards.

**Thank you for your comments.**

35. Reviewer #2: I read the manuscript by Nigel Franks et al. with great interest. The authors present a comprehensive study of post-contact immobility in predatory behaviour of antlions. The study is a very interesting paper, containing valuable new information, worthy of publication. I suggest to accept it after minor revision.

**Thank you.**

36. Comments and suggestions:

Lines 72-74: Antlions are also prey by conspecifics. It would be wise to mention immobility in such cases. See also a paper by Klokocovnik et al. 2020: Klokočovnik V., Veler E., Devetak D. 2020. Antlions in interaction: confrontation of two competitors in limited space. Israel Journal of Ecology & Evolution, 66 (1-2): 73-81. DOI: 10.1163/22244662-20191058

**Thank you very much for alerting us to the interesting phenomenon investigated in this paper. We could not find an appropriate place to cite it in our current manuscript without breaking the flow or being perfunctory and we already have more than 70 references. However, we will keep it in mind in the future.**

37. Lines 152-153: "Hard or soft substrates" - These terms refer to density. The following paper could be cited here: Devetak D., Novak T., Janžekovič F. 2012: Effect of substrate density on behaviour of antlion larvae (Neuroptera: Myrmeleontidae). Acta Oecologica, 43: 1–7. <http://dx.doi.org/10.1016/j.actao.2012.05.010>

**Again, thank you very much for alerting us to this publication. It is more focused on pit-building behaviour, which is an extensive area of study, and we do not think we can make a natural brief connection with our present study any more than with other studies in this fascinating area.**

38. Lines 610-612: Here you should cite also a review paper dealing with the role of the sensory hairs in antlions: Devetak, D. 2014. Sand-borne vibrations in prey detection and orientation of antlions. In: R.B. Cocroft, M. Gogala, P. S. M. Hill, A. Wessel (eds.): Studying Vibrational Communication. Animal Signals and Communication 3, DOI: 10.1007/978-3-662-43607-3_16. Berlin, Heidelberg: Springer-Verlag, pp. 319-330.

**Thank you. We have managed to find a pdf of this interesting and relevant reference. It has been cited as reference [31] in the revised manuscript (L. 156 and L. 623).**

39. Reviewer #3: Dear Authors, I have reviewed the manuscript “Seeking safety: movement dynamics after post-contact immobility” by Franks et al. for possible publication in journal PLOS ONE. Authors have investigated the post contact mobility/immobility after in antlion larvae after post contact immobility induced by authors. The article is well written, a bit lengthy in certain parts. The topic is interesting as post contact immobility is an important behavioural pattern, which is still not sufficiently explored, especially what happens after PCI.

**Thank you.**

40. The topic and the concept of the experiment is very interesting. The quantitative approach used for analysis of this phenomena has quite some potential. However, there are some major issues regarding the methodology and experimental procedure.

**Thank you. Please see how we have addressed the specific comments by Reviewer #3 under comments #41 to #55 below.**

41. One problem is the temperature range in which the experiment has been executed. There are several studies that are concerned with the impact of temperature on physiology and behaviour of insects, even on antlion larvae. Five degree (5 °C) difference could influence the duration of immobility/mobility between replicates. Another problematic aspect of this study is the sample size (see the remarks below).

**Crucially, the variation in temperature is not conflated with the variation in movement/immobility durations on different substrate types. The range 20 to 25 ^o^C (L. 176) refers to variation throughout the day (L. 176) and we conducted two sessions for each experimental treatment, one in the morning and one at midday or early afternoon. We have edited the text to read “Experiments were carried out in two sessions (one in the morning and one at midday or in the early afternoon, to cover the temperature range, S1 Table) …” (LL. 184-185). In addition, the temperature range in our experiments is similar to what could be found in the literature: for example, 22 to 26 ^o^C (Mencinger-Vračko, B. and Devetak, D., 2008. Orientation of the pit-building antlion larva Euroleon (Neuroptera, Myrmeleontidae) to the direction of substrate vibrations caused by prey. Zoology, 111(1), pp.2-8.)**

42. Comparing movement on hard surface and in the sand is quite challenging task in antlion larvae, as antlions use one substrate for burrowing and hiding (their natural microhabitat), while hard surfaces are inappropriate substrate for sand dwelling species and it rushes to find cover as soon as possible. This is reflected in the results of this study. We do not know if the results would differ if antlion larvae would be placed gently on the surface instead of being dropped. So the question is, are we observing the effect of PCI induced by different substrates (hardness) or simply the effect of different substrate on antlion larva, which could be the same even if the animal is not dropped on the surface.

**There seems to be a misunderstanding here. If placing the antlion larvae gently on the surface involved enough contact to induce PCI, the experiment could have been very similar. However, inducing PCI by dropping is a procedure that is straightforward to standardise and was intended to reproduce the action of a bird. Please see our benchmark study, [9], and references therein. On the other hand, if placing the antlion larvae gently on the surface did not induce PCI, the experiment would not have been what we intended. If we had not simulated the actions of a putative predator and induced PCI, there would not have been a trade-off in the antlion response between staying still to avoid immediate predation and moving to hide from future danger. Hence, we would expect different results.**

43. Unless authors are able to properly address these issues I do not think that the manuscript is acceptable for publication in PLOS ONE.

**Please see our responses under comments #41 and #42 above.**

44. Other remarks

L55: Consider adding the term thanatosis, which is widely used.

**The term ‘thanatosis’ has been added (L. 55).**

45. L134-151: Part of this paragraph should probably be in the method section. Retain only enough information for the purpose/goal of the experiment remains clear.

**We would like to keep the whole of this paragraph in the Introduction because it explains our approach to addressing the questions we pose in the context of earlier work.**

46. L167: Small sample size, probably too small (especially for both sand experiments).

**In terms of power, our sample size was large enough to show an effect of the substrate. Hence, the biological signal must be strong. In terms of representativeness, the population of this species is small on the British Isles, including the one we sampled in Guernsey. We also used a repeated design. Smaller samples and a repeated design are encouraged when studying small populations. This is represented in the Reduction principle of the 3Rs (**[**https://www.nc3rs.org.uk/who-we-are/3rs**](https://www.nc3rs.org.uk/who-we-are/3rs)**).**

47. L169-171: There is quite a range in animal weight. You probably used different instars. You should provide information: which instars were used in the experiment and number of individuals from each instar. There is evidence about difference in behaviour/activity of different instars (Alcalay et al. 2014 – article cited below). Did you notice any difference in PCI and post PCI behaviour in regard to the size of animals?

**In response to comment #27 by Reviewer #1 above, we have edited this sentence to read “Larval weight covered a wide range and likely represented the 2^nd^ and 3^rd^ larval stages (median = 0.0360 g, LQ = 0.0183 g, UQ = 0.0850 g, S1 Table) (LL. 173-175)”. In fact, the distribution of weight (g) fell in three non-overlapping groups: mean weight (g) ± standard deviation for larvae: first group, 0.0156 ± 0.00534 g (n = 8); second group, 0.0365 ± 0.00383 g (n = 6), third group, 0.0931 ± 0.01430 g (n = 8). However, we are not convinced these groups map on the three larval instars and hence have not included this information in the text. We found no effect of antlion weight in our models in both the present and earlier studies of movement and immobility (LL. 304-306).**

48. Additionally: you give the mass of median, LQ and UQ with more precision than your measurements allow. Please correct it.

**This is intentional because we follow the rule-of-thumb that summary statistics like the mean and the median are reported with one more decimal place than the precision for raw measurements (Zar, J. H. 1999. 4^th^ Edition. Biostatistical Analysis, Prentice Hall, New Jersey, p. 79, Example 6.5). Of course, we are happy to round all the reported values to the third decimal place if the Editors at PLoS One advise us that this is the journal style we need to follow.**

49. Alcalay Y, Barkae ED, Ovadia O, Scharf I. Consequences of the instar stage for behavior in a pit-building antlion. Behav Processes. 2014 Mar;103:105-11. doi: 10.1016/j.beproc.2013.11.009. Epub 2013 Dec 4. PMID: 24315799.

**Thank you for the reference to this study. It shows dependence of behaviour on the larval instar in the context of pit building and in a different antlion species. Pit building is a fascinating topic but we did not find an effect of weight in the context of PCI and substrate.**

50. L171-172: Temperature variation was 5 °C, which is high, especially when analysing parameters like immobility and mobility duration. There are several papers on this topic.

**Please see our response to comment #41 above.**

51. L188-192: What was the substrate particle size?

**Please see our response to comment #31 above.**

52. L191-193: This an unusual formulation. A “typical antlion larva”? I believe there is no such thing. There are 3 instars of antlion larvae. They differ substantially in size. You should rephrase.

**We rephrased the sentence to read “A typical *E. nostras* 3^rd^ instar larva …” (L. 200). In addition, we would like to apologise for the error in the height: it should read 4.4 mm, not 3.4 mm (L. 200). This has now been corrected.**

53. L193-194: For a small first instar larva the 2 mm of sand should be enough to cover/burry itself with sand. Therefore, it is important, as stated before, that you provide information about the instar of your individuals and number of individuals from each instar.

**Please see our responses to comments #27 and #47 above.**

54. L212-216: Even though, authors justify the small sample size (especially for the sand substrate experiment) with experimental design and use of appropriate statistical method, I question if 8 antlion larvae (even 5 in one case) of different mass and larval stage can be representative sample for making strong conclusions.

**The population we sampled is a small one and the 22 antlion larvae used in the study were collected in an as unbiased way as possible. The 8 antlions for each of the two sand substrates was chosen as a stratified random sample from the original 22 individuals in relation to weight (LL. 213-215). Hence we think that our results are representative.**

**The sample size of 5 refers to the number of antlion larvae with the full suite of 16 pairs of immobility and movement periods on Deep sand. The remaining 3 antlions in this treatment had 12, 8 and 13 pairs of immobility and movement durations simply because they buried themselves. This result only comes to confirm our findings that there was less movement on Deep sand than on Shallow sand, where all 8 antlion larvae had the full set of 16 pairs of immobility and movement periods (LL. 247-250).**

55. L285: As pointed out before, the sample size is important for drawing conclusions from obtained results. I suggest additional reviewer skilled/competent in statistical methodology.

**Sample representativeness depends on the size of the sample relative to the population size and unbiased sampling. We argue that our sample is representative on both these counts. Flipping the argument the other way around, a large sample is not necessarily representative and could actually overstate the case for only a small biological difference because with a large enough sample any difference would be significant. Hence, in modern biology one should not take larger samples than needed and should state effect sizes as well as p-values.**

56. Reviewer #4: The authors examine the pattern of movement and immobility when antlion larvae resume activity after Post-Contact immobility (PCI). To simulate contact with, and escape from a predator they dropped the larvae onto three substrates: paper, shallow sand, and deep sand. The authors tracked the antlion's paths automatically and recorded alternating immobility and movement durations over 90 minutes. Their results suggest that post-PCI movement and immobility gradually return to the pattern typical of intermittent locomotion, depending on the scope for burying and hiding in the substrate.

**Thank you for this clear summary of our study.**

57. I found the topic of this manuscript interesting. The work also shows an adequate experimental design (despite the low number of replicates and the use of paper instead of other more natural substrates, see below), and is relatively easy to read (despite the great relevance given to mathematics and statistics instead of biology, see below). I have only a few comments that I hope help to improve this nice manuscript.

**Thank you for the encouragement.**

58. First, my feeling is that the authors try to calculate the area of a square by integrating instead of multiplying their sides. This metaphor intends to explain my sensation about the use of complex statistical and math tools to demonstrate what seems a predictable and simple behavior: after PCI, in hard and shallow substrates, antlions move to look for a better place to dig, but in deep sand, antlions move mainly to buried themselves. This does not invalidate the interesting and novel topic of the manuscript, but –in my opinion, the authors take a complicated way instead of a simple one to reach a biological conclusion that is relatively predictable.

**We are glad Reviewer #4 finds our results as what is to be expected and yet novel. To continue with the metaphor they use, there is an argument that “integrals are more important than areas” because “areas are crucial to geometry” while “integrals are crucial to *everything*” (Strogatz, S. 2019. Infinite Powers. Atlantic Books, London, p. 181). In any case, we have used standard statistical methods (general linear mixed models and the standard method for analysing distributions of immobility and movement duration). The automatic image-analysis tracking of antlion locomotion on the sand substrates was challenging even with the white sand used to maximise contrast.**

59. Second, I think that dropping antlion on a stone surface would have been a much better –and real- option than dropping them on a paper surface. I understand that this experimental design can no longer be modified, but the use of paper instead of stone should be better justified.

**We would like to emphasise that the paper was lining the bottom of the experimental plastic Petri dish (LL. 134-135). Therefore, it represented a hard impenetrable surface, which probably was not as hard as a stone but was much harder than sand. In any case, the bristles on the bodies of antlion larvae will soften the fall impact as much on a stone surface as on paper lining the bottom of a Petri dish.**

60. Third, the authors predict that antlions will search for the relative safety of escaping altogether from a hard substrate since they cannot hide by digging. I agree, but ¿there are other options? In other words, if escape from this substrate is the only alternative, this result does not necessarily support the hypothesis that generates it. I believe that it is necessary to clarify if different alternatives exist and why escape is the one that is an unequivocal consequence of the hypothesis.

**The way of escape provides alternatives. In advance of carrying out the actual experiment, one could predict that the antlion larva would somehow balance the danger from the putative predator that has just dropped them (moving immediately) against other predators and dangers (not moving at all). However, it is difficult to predict how. That is why our analysis of the change of immobility and movement durations after the induction of PCI is important. We have added a sentence along these lines to the revised manuscript. So this section now reads “We predict that they search for the relative safety of escaping altogether from a hard substrate, since they cannot hide by digging. The question is how they balance the immediate danger from the putative predator that has dropped them against the future danger of being exposed on the substrate surface.“ (LL. 161-163).**

61. L. 126. Farji-Brener (2003) could be an adequate reference here. Reference Farji-Brener, A. G. (2003). Microhabitat selection by antlion larvae, Myrmeleon crudelis: effect of soil particle size on pit-trap design and prey capture. Journal of Insect Behavior, 16(6), 783-796.

**Thank you. We have added this reference as [26] (L. 126).**
