# Supplementary material for: Seeking safety: Movement dynamics after post-contact immobility
Source: PLoS One. 2024 Aug 22;19(8):e0307370. doi: 10.1371/journal.pone.0307370 (PMC11340899; doi:10.1371/journal.pone.0307370)
Supplement: Attachment — Submitted filename: Response to Reviewers PONE-D-23-29142R1.docx [file pone.0307370.s017.docx]

**PONE-D-23-29142R1**

**Seeking safety: movement dynamics after post-contact immobility**

Additional Editor Comments:

1. After this new review round, both reviewers recognize you have reached a significant improvement in your manuscript, from the original version to the newly submitted version. One of the reviewers is satisfied with the new version and approved its acceptance. Still, the other reviewer is still concerned about the overall design of the experiment. The first issue regards the temperature range used in the experiment. According to their view, a 5-degree range significantly affects the insect's behavioral repertory, which I also tend to agree with.

**Thank you for agreeing to handle the review of our manuscript. In fact, the temperature range is not mentioned by Reviewer #3. However, in the earlier round of the review process, we responded to comments about the temperature range – see next paragraph – and we have now also cited the paper by Mencinger-Vračko and Devetak (2008) in the revised manuscript (L. 178).**

**“Crucially, the variation in temperature is not conflated with the variation in movement/immobility durations on different substrate types. The range 20 to 25 ^o^C (L. 176) refers to variation throughout the day (L. 176) and we conducted two sessions for each experimental treatment, one in the morning and one at midday or early afternoon. We have edited the text to read “Experiments were carried out in two sessions (one in the morning and one at midday or in the early afternoon, to cover the temperature range, S1 Table) …” (LL. 184-185). In addition, the temperature range in our experiments is similar to what could be found in the literature: for example, 22 to 26 ^o^C (Mencinger-Vračko, B. and Devetak, D., 2008. Orientation of the pit-building antlion larva Euroleon (Neuroptera, Myrmeleontidae) to the direction of substrate vibrations caused by prey. Zoology, 111(1), pp.2-8.).”**

2. Another issue is that the reviewer believes comparing Antlion's movements on both hard/sand surfaces is also troublesome and should affect the experiment's results, which I also think is an important issue that may affect the obtained results.

**There is a misconception here. Please see our responses to comments #6 and #7 below.**

3. Finally, other minor improvements are suggested and (as far as I can see) should be addressed also in the next version of the manuscript.

**Please see our response to comment #9 below.**

4. In my experience as an editor and working with insect behavior a long time ago, I consider you would have two paths to solve the issues raised by the reviewer: one that may seem more "easy" but that may prove to be fatal and a more difficult one. The "easy" one would be to add information on how antlion experiments similar to yours are made, explaining how the general research with these insects in your field of research is made and how your experimental design complies with the other studies, showing there was no fatal flaw with the methods you employed. The second and more difficult one would be to set up a new experiment, showing if different manipulation strategies and temperature ranges do not affect the results you obtained. If the result of the experiment considering different manipulation strategies and temperature ranges does not show any significant difference among the considered variables, I believe the reviewer will (hopefully) provide their blessings to your manuscript and accept it for publication. I want to stress that this is also possible by following the "easy" path. Still, please note that in such a path, in the end, the reviewer may not agree with what has been argued and may end up rejecting the manuscript.

Since this is not an easy choice, I will provide you with a four-month period (June 15th, 2024) to deliver the new version of the manuscript. If you decide to follow the more difficult path, I believe you will have enough time (I think) to plan this new experiment. Still, in case you decide on a difficult path and still need more time, please let me know, and I will grant you more time to complete the decided task. Finally, I would like to acknowledge the efforts made by the reviewers and I hope to count on their knowledge in the next version of the study.

**Thank you. The request for additional experiments is not justified (please see our responses to Reviewer #3’s comments below). We too are grateful to the referees for their repeated efforts with reviewing our work. Reviewer #4 was satisfied and approved acceptance while Reviewer #3 was not satisfied about the experimental design, a point we rebut robustly below. It is the editor’s duty to weigh the arguments equitably and the declaration that our paper can only be published when the second referee is satisfied is an abrogation of this duty.**

Reviewer #3:

5. Dear Authors, I have read the revised manuscript “Seeking safety: movement dynamics after post-contact immobility” by Franks et al. Thank you for extensive clarifications of comments given in previous round of review. Most clarifications are satisfactory. I still believe that the manuscript has some merit, especially in methodological approach. However, there is still one issue I am concerned with. Same issues were raised by other reviewers as well (except one) and your answer did not satisfy me.

**Thank you for your repeated efforts on our manuscript. Please see our responses below.**

6. Authors explore what is the course of events in a potential prey after it was stressed, dropped on the surface, which induced a thanatosis state, a behavioural mechanism for avoiding predators when hiding is not possible. While dropping animals on different substrates induces PCI of different duration (not statistically significant in this study), what happens after this behavioural pattern ends, is also a function of very simple reflex behaviour of an animal that submerges into substrate when this is possible and seeks a place to submerge when this stereotypic behaviour is not an option. This means that it is not clear if the induction of PCI has a crucial contribution to events that happen after it – the events this study is exploring. It could be a normal response of an antlion larva to the different substrate regardless of before induced PCI. The experiment is lacking a control, which would validate this part of author’s hypothesis.

**Using antlions without inducing PCI is not an appropriate control for our experiments. In the absence of PCI, an antlion has to find cover as quickly as possible while in the presence of PCI, an antlion has to balance the necessity to find cover as soon as possible with the immediate danger of being spotted by the putative predator that dropped it and thus induced PCI. The antlion is in an emergency in both cases, but the best strategy to deal with the emergency is different. Therefore, placing antlions on substrates without inducing PCI is a different treatment, not a control. An appropriate control is the known duration distribution for immobility/movement in animals of diverse taxa in a neutral environment. That is the control we have used. A neutral environment for an antlion larva is deep in the sand. Indeed, our results show that the patterns of movement and immobility and their duration distributions were approaching the control in the deep-sand treatment, just about deep enough for submergence but even at these relatively shallow depths it was challenging to track the antlions.**

7. The second problem I see is the comparison of movement on hard surface and in the sand. Antlions use one substrate for burrowing and hiding (their natural microhabitat), while hard surfaces are inappropriate substrate for sand dwelling species, and it rushes to find cover as soon as possible. This is reflected in the results of this study – predictable outcome, as hard surface does not offer anything else than looking for place to bury itself. You say: “The question is how they balance the immediate danger from the putative predator that has dropped them against the future danger of being exposed on the substrate surface. “ So the answer is: They burry them self if possible – sand substrate -, if not, they use a certain pattern of immobility and mobility, which is an answer to the trade-off you mention. But you do not know if this pattern is any different if no PCI is induced before.

**There is no reason for the pattern of movement and immobility to be the same if PCI is not induced. As Reviewer #3 states, if PCI is not induced the antlion rushes to find cover while if PCI is induced, it faces a trade-off. The solution to this trade-off depends on the substrate. For example, when antlions come out of PCI on the hard surface, their successive immobility durations get gradually shorter than that of PCI and their movement durations get gradually longer. When antlions come out of PCI on shallow sand this relationships are weaker and on deep sand they do not exist. By contrast, if PCI is not induced an antlion has to find cover as soon as possible and its movement durations should not depend on time on any of the three substrates (though there might be other differences between the substrates, in mean movement duration, for example). Therefore, for one thing, the gradation in the pattern of movement and immobility from the hard surface, through the shallow sand to the deep sand (Figs. 1 & 2) is consistent with the expected trade-off when antlions come out of PCI. This gradation does not make sense in the absence of PCI when antlions need to submerge or take cover as soon as possible.**

8. Because of these conceptual issues I still have concerns about the manuscript suitability for publication in PLOS ONE Journal.

**The issues raised are based on a misconception and we have rebutted them. Our work is novel and has implications about PCI beyond antlions. Indeed, Reviewer #4 thinks our paper is ready to be published.**

9. Minor comments:

L134-151: I still believe that a big part of this paragraph belongs in the method section. Retain only enough information for the purpose/goal of the experiment remains clear or it becomes even clearer.

**We would still like to keep the whole of this paragraph in the Introduction because it explains our approach to addressing the questions we pose in the context of earlier work.**

Reviewer #4:

10. I just read the second version of the paper entitled “Seeking safety: movement dynamics after post-contact immobility” In general, I found this version clearer than the previous one. I believe that this interesting paper is ready to be published. I only have minor comments regarding my previous suggestions.

**Thank you.**

11. First, I still feel that the use of complex statistical and math tools to demonstrate what seems like a predictable and simple behavior is not the best way. I am glad that the authors follow my metaphor about how is the better and simpler way to calculate the area of a square, and I fully understand that “integrals are more important than areas because areas are crucial to geometry, while integrals are crucial to everything” But it is also true that simpler analyses are “easier to explain and understand; they clarify what the key units in a study are; they reduce the chances for computational mistakes; and they are more likely to lead to the same conclusions when applied by different analysts to the same data” (Murtaugh 2007). Maybe is an interesting topic to discuss in other contexts (e.g.; drinking a beer) rather than here.

**Thank you for the reference. Yes, we agree with the principle of making quantitative analyses as simple as possible. Perhaps, where we disagree is in whether we could have had simpler analyses in the present study. We have used the standard linear models commonly applied in animal behaviour and ecology. We had to include the random factor because of the repeated design with the same individual antlions (a recommended design). The analyses of distributions is also the standard of its kind. Both analyses are repeatable through commonly used R packages.**

12. Second, I also understand that the paper lining the bottom of a plastic Petri dish represents a hard impenetrable surface, but this scenario is not the best representation of what happens in nature. The authors could discuss and make clear what real scenario this lab condition tries to represent (e.g. when larvae are drooping on compact soil, a stone surface, or on a fallen trunk) and argue their eventual limitations.

**We do, in fact, mention the stone surface scenario (LL. 132-133). To make this even clearer, we have edited the start of the next paragraph to read “We induced PCI on three different substrates: (1) Paper, lining a Petri dish, representing an impenetrable hard surface, such as the stone surface on which the predator might have inadvertently dropped the antlion; …” (LL. 134-136). This is, of course, an experiment and, as such, is a controlled representation but not a description of what happens in nature.**

13. Finally, if the substrate is so hard that impedes the larvae buried themselves, there are no other expected results than keeping immobile or moving looking for a better place to be buried. I believe that it is necessary to clarify if different alternatives exist and why escape is the one that is an unmistakable consequence of the hypothesis.

**We agree that there is no alternative to looking for cover on the hard surface. However, there are different ways of doing it in terms of the pattern of movement and immobility after PCI. We have added a sentence along these lines to the revised manuscript. This section now reads “We predict that they search for the relative safety of escaping altogether from a hard substrate, since they cannot hide by digging. The question is how they balance the immediate danger from the putative predator that has dropped them against the future danger of being exposed on the substrate surface. So we ask what is their pattern of mobility and immobility after PCI? (LL. 161-165).”**

14. Congratulations for your nice work!

**Thank you.**
