# Supplementary material for: Seeking safety: Movement dynamics after post-contact immobility
Source: PLoS One. 2024 Aug 22;19(8):e0307370. doi: 10.1371/journal.pone.0307370 (PMC11340899; doi:10.1371/journal.pone.0307370)
Supplement: Attachment — Submitted filename: Response to Reviewers PONE-D-23-29142R2.docx [file pone.0307370.s018.docx]

**PONE-D-23-29142R2**

**Seeking safety: movement dynamics after post-contact immobility**

Additional Editor Comments:

After this new review round, a new reviewer still raised significant issues that need to be considered in the new version of your text. The reviewer that accepted the MS made similar reservations regarding the text to me, letting me decide the fate of your manuscript. Since I am not an expert in the topics covered in your text, and since two experts reached the same decision, although one let me decide if I should accept it or not, I believe the correct decision to make is to be able to convince reviewer #5. See, the amount of issues raised is considerable and, at this point, I believe I could only accept the text to be published if, in the next version, reviewer #5 believes the text deserves minor reviews from there on. Therefore, I will grant you a new 2-month period to deliver your manuscript and rebuttal letter.

**Thank you for handling our manuscript. We are also grateful to the two reviewers for their time and efforts, particularly to the new Reviewer #5 for their detailed review. We have addressed all the comments by Reviewer #5 in our point-by-point list of responses below. We are confident that the Editor will weigh equitably the arguments made by reviewers and authors. Reviewers could make mistakes and be unreasonable as could authors.**

Reviewer #5:

1. Dear Authors, I have now read the manuscript titled “Seeking safety: movement dynamics after post-contact immobility” (PONE-D-23-29142R2). In this study the authors investigated the defensive behavior of an antlion after being dropped by a surrogate predator on different surfaces (deep sand, shallow sand and paper). The paper is overall well written, and the research questions are relevant. Moreover, the authors advocate the importance of using quantitative approaches in post-contact immobility (AKA tonic immobility, death feigning, thanatosis, to name a synonymous). This suggestion is among the highlights of this manuscript, and the literature on the topic would greatly benefit from better understanding the quantitative properties of this behavior across animal taxa. Even though the manuscript is of good quality, I have a few major concerns that are listed below (by section) followed by minor comments/suggestions:

**Thank you for this encouraging summary of our work.**

Major comments

2. Experimental design:

- While the treatment and controls chosen are appropriate to answer the chosen biological question, I am afraid that a few aspects of the current design and their possible effects on the results deserve further attention:

**Please see our responses to each of the specific points under comments #3-#8 below.**

3. 1-The authors did not standardize the feeding state of the experimental models: It is well documented in the scientific literature that the level of starvation can affect the behavior of arthropods. Please see Scharf (2016). Anim Behav, 119, 37-48 and references therein. As such, one cannot rule out the fact that by chance individuals bearing different levels of starvation may have been disproportionally chosen for different experimental groups. Especially given the relatively low sample size used in this study (see topic below). Therefore, the results may have been confounded by the non-standardized body state of the experimental models.

**Such disproportionate representation of feeding state is very unlikely because we selected the eight antlions for each of the Shallow Sand and Deep Sand treatments as a stratified random sample from the 22 antlions we collected after sorting them according to weight (LL. 220-225). Please see also our response under comment #15 below. In addition we carried out the following test. We assumed that individuals with higher levels of starvation were disproportionately represented in one of the two groups of eight tested on sand (Shallow or Deep) and that any differences between these two groups were explained by the level of starvation rather than the type of sand substrate.** **Therefore, we expected the eight individuals tested on Shallow sand to differ in their behaviour from the eight individuals tested on Deep sand when all 16 were tested on Paper. We found no such difference either for immobility or movement duration (corresponding to Figs. 1 and 2 in the manuscript, respectively). Please see below the respective plots and results from statistical analyses. Hence, we conclude that one of the following is true: (a) antlions with higher starvation levels were not disproportionately represented in one of the two groups tested on sand, (b) starvation level did not affect the studied antlion behaviour, (c) starvation level affected the behaviour only on the sand substrates but not on Paper (a very remote possibility). We have included a point to this effect in the revised version of the manuscript (LL. 604-612).**

**lm(log10(Idur_s) ~ log10(Iseq_no) + TreatPbyS + log10(Iseq_no):TreatPbyS, data = d_iall)**

**Estimate Std. Error t value Pr(>|t|)**

**(Intercept)	2.35820	0.24118	9.778 < 2e-16 ***	Intercept for Paper after Deep sand**

**log10(Iseq_no)	-1.45466	0.38372 -3.791 0.000245 ***	Slope for Paper after Deep sand**

**TreatPbyS2.3mm sand	-0.15359	0.33589 -0.457 0.648390	Difference between intercept for Paper after Deep sand and intercept for Paper after Shallow sand, NS**

**log10(Iseq_no):TreatPbyS2.3mm sand	-0.02969	0.51685 -0.057 0.954293	Difference between slope for Paper after Deep sand and intercept for Paper after Shallow sand, NS**

**log10(Mdur_s) ~ log10(Mseq_no) + TreatPbyS + log10(Mseq_no):TreatPbyS, data = d_mall)**

**Estimate Std. Error t value Pr(>|t|)**

**(Intercept)	-0.01544	0.16383 -0.094	0.9251	Intercept for Paper after Deep sand**

**log10(Mseq_no)	1.19707	0.26066	4.592 1.17e-05 ***	Slope for Paper after Deep sand**

**TreatPbyS2.3mm sand	-0.41458	0.22817 -1.817	0.0719 .	Difference between intercept for Paper after Deep sand and intercept for Paper after Shallow sand, NS but p-value small, just above 0.05; however intercept not meaningful since measurement precision was 1s.**

**log10(Mseq_no):TreatPbyS2.3mm sand	0.36884	0.35110	1.051	0.2958	Difference between slope for Paper after Deep sand and intercept for Paper after Shallow sand, NS**

4. 2-The samples size are strongly unbalanced. While the experimental group tested with paper subtract had a relatively acceptable sample size (N = 22), for the both the groups tested with subtracts Deep and Shallow, one could argue that the sample size was too low (N = 8), especially considering the high variance typically found in behavioral studies. With that being said, I am afraid that this unbalance led to an augmented chances of incurring in an error type I [see Forstmeier et al (2017). Biol Rev 92,1941-1968]. The authors made use of bootstrapping to deal with this issue, but resampling the measures may not be enough if the collected data does not provide correct estimates of the underlying population.

**The main point made by Forstmeier et al. (2017) with regard to small sample size concerns the False Positive Report Probability (FPRP) in a situation where there are a lot of hypotheses to be tested and only a small proportion of them is true.** $\boldsymbol{FPRP=}\frac{\boldsymbol{\alpha}\left( \boldsymbol{1-\pi} \right)}{\boldsymbol{\alpha}\left( \boldsymbol{1-\pi} \right)\boldsymbol{+(1-\beta)\pi}}$, **where** $\boldsymbol{\pi}$ **is the proportion of true hypotheses among those to be tested, α is the probability of Type I error (false positive result, rejecting Ho when it is true, equivalent to the significance level, usually set at** $\boldsymbol{0.05}$**,** $\boldsymbol{\beta}$ **is the probability of Type II error (false negative result, failing to reject Ho when it is false),** $\boldsymbol{1-\beta}$ **is the power of the test (the probability of rejecting Ho when it is false). The main point is that** $\boldsymbol{FPRP}$ **is high when the power** $\boldsymbol{1-\beta}$ **is low. For example if** $\boldsymbol{\pi=0.10}$**,** $\boldsymbol{FPRP=0.36}$ **when** $\boldsymbol{1-\beta=0.80}$**, but** $\boldsymbol{FPRP=0.69}$**, when** $\boldsymbol{1-\beta=0.20}$**. In both cases, but particularly when the power is low,** $\boldsymbol{FPRP}$ **is considerably higher than the usual significance level,** $\boldsymbol{\alpha=0.05}$**. However, this is of concern only when the proportion of true hypotheses** $\boldsymbol{\pi}$**, is very low, as in the preceding example. By contrast, if** $\boldsymbol{\pi}$ **is high, for example, if** $\boldsymbol{\pi=0.90}$**,** $\boldsymbol{FPRP=0.01}$ **when** $\boldsymbol{1-\beta=0.80}$ **and** $\boldsymbol{FPRP=0.03}$**, when** $\boldsymbol{1-\beta=0.20}$**. In other words, when 90% of the hypotheses to be tested are correct,** $\boldsymbol{FPRP}$ **does not fall below the usual significance level,** $\boldsymbol{\alpha=0.05}$. **Now, the** $\boldsymbol{FPRP}$ **is relevant when applied to meta analyses of the results of many papers or to individual papers with many sequentially tested hypotheses. We are not testing many hypotheses. Furthermore, the** $\boldsymbol{FPRP}$ **focuses on positive reports, namely on cases where Ho has been rejected (rightly or wrongly). It ignores the cases with failures to reject Ho when it is false, the probability of which is** $\boldsymbol{\beta}$**, Type II error. This is a much more relevant limitation of our study and exploratory studies in general. It limits what we might have been able to show. However, in other countries, populations of antlions of the same species are not rare and small as on the British Isles and we hope future studies could demonstrate differences that we have not been able to show. We have added a point to this effect in the revised manuscript (LL. 592-603).**

5. 3-Given that the group “paper substrate” had a significantly larger sample size than the other two “Deep and Shallow Sand”: I wonder if the time of the test might not have influenced disproportionally the experimental groups? Additionally, given the repeated measures experiment, I would request the authors to provide a file with the order of the tests (a sequence informing the order in which each sampled individual went through concerning the experimental groups and time of the day when they were tested)

**The experiments with the Shallow sand and Deep sand treatments were carried out on 14^th^ June and 15^th^ June 2019, respectively. The Paper treatment was carried out over 16^th^ to 18^th^ June 2019 (S1 Table). The conditions in the room where we carried out the experiments were similar over these five experimental days and all three treatments included two sessions: one in the morning and one around lunch time (to cover the temperature range, LL. 187-188). In addition, PCI duration is reproducible in these species when measured again within 1 to 3 days (LL. 242-243). Therefore, there is no reason to suspect that the time of the test might have influenced disproportionately the experimental groups. We have added a point to this effect in the revised manuscript (LL. 239-248). Please also see our responses to comments #16 and #17 below.**

**In fact, the current S1 Table does provide the order of tests, including information on each individual, treatment, time of day, date, the quadrant in the 2x2 grid of four Petri dishes and the order of delivery given that antlions were delivered to the substrate from left to right on each row, starting with the first row (LL. 217-219). For example, Antlion 1 underwent the Deep sand treatment on 15^th^ June 2019 in the second session and was delivered last (4^th^).**

6. Discussion:

The discussion section of the manuscript would greatly benefit from making comparisons of the findings of their study with similar results from the literature showing defensive behavior to be context dependent [see also Threat Sensitive Hypothesis, e.g. Gyssels & Stoks (2005). Ethology, 111: 411-423], especially in “post-contact immobility” for example, concerning daily cycle [Jones et al (2011). Anim Behav 82:549–555; Miyatake (2001). J Insect Behav 14:421–432; Segovia et al (2019). J Arachnol, 47:396-398; Watts et al (2014). Anim Behav 94:79– 86].

**Thank you. We have done that and have now cited two of the above studies (LL. 628-636).**

7. I struggle with the idea of assuming that a pattern of behavior is evidence of distress on its own. When a given behavior is associated with an objective mechanistic measure of stress (e.g. cortisol levels) in the very same species, one might speculate this behavior to be a proxy of distress yet incurring in some risk. However, if that is not the case, one could call whatever different behavioral pattern emerges a measure of distress, and I suspect that it is not the most parsimonious approach. As such, I would suggest the authors to either rethink the use of this term in the manuscript or build a more solid argument on why that behavior is a valid measure of distress (if that is the case).

**We have changed ‘distress’ to ‘disruption’ or ‘disruptive event’ (LL. 45, 113-114, 116, 625, 653, 662). Earlier we used the term ‘distress’ as per reference [21] on the basis of observing quantitative patterns in the movement and immobility of antlions that are consistent with those observed in other species when individuals are in a distressed or an impaired state [21 and references therein]. In some of the literature cited an association has been established between such a distressed or an impaired state and the presence of exogenous and endogenous stressors. We hope our results will encourage physiologists to look for such associations in the aftermath of PCI in antlions too.**

8. Additionally, it would be desirable to include a paragraph in the discussion section about the possible limitations resulting from the experimental design (Comments 1-3, inexperimental design section above)

**We have added to the text in response to each of the three above comments on experimental design. For details, please see our responses to comments #3-#5 above.**

Minor comments:

9. Theoretical approach:

L 177: Please provide metadata for S1Table.

**To the best of our understanding, we have provided the information about the data in S1 Table in its caption (LL. 923-938) and hence request an example of what is deemed to be missing.**

10. L 186-187: Were the effects of time and temperature added as random effects in the models? If possible, the readers could benefit from having access to the scripts with the fitted models.

**The experimental date corresponded exactly to the treatment (please see our response under comment #5 above) and hence it would not make sense to include it in the model****. Session 1 or 2 (morning or around midday) was added as a random factor. The resulting model for both immobility and movement duration had 0 variance and AIC that was 2 points higher than the best model for the respective response variable with Chi-sq = 0, df = 1, p = 1. This result is corroborated by the boxplots below. Hence we kept the original best models. We have included a point to this effect in the revised manuscript (LL. 328-331).**

**Yes, for the benefit of readers we will include the scripts with the fitted models on acceptance of the manuscript for publication.**

11. L 191-193: Please clarify.

**The camera we used had the advantage of higher spatial and temporal resolution but also the downside of a limit of 22 min 31 s duration for continuous filming. Hence, we had to restart it. We have added this clarification to the text (LL. 193-195).**

12. L 201: Have you used the same sand described above?

**Yes, we used the same make of white decorative sand for the Deep sand treatment as for the Shallow sand treatment. We have now made this explicit in the text (LL. 204-205).**

13. L 202: Please provide the data (a measure of central tendency and deviation) concerning the size of the experimental models, the best case would be if you could provide it by experimental groups.

**Our measure of the distance between the highest point on the thorax and the lowest point on the coxa of the third leg of a typical *E. nostras* 3rd instar larva is based on a specimen’s photo with a scale in [36]. We used this estimate to gauge the approximate depth of sand that will cover most of the experimental antlions partially or just over the top for the Shallow sand and the Deep sand treatment, respectively.**

14. L 205-208: Could you please clarify it? It would be done even by rephrasing or providing a schematic picture.

**We have rephrased this section and hope it is now clear that** **for each antlion in each treatment and session the substrate surface was fresh (LL. 208-213).**

15. L 215-207: Shall you please be more specific about the criteria adopted to distribute the larvae according to the weight (experimental groups? Time?).

**We have elaborated this point and hope it makes clear how we used stratified random sampling to avoid any disproportionate representation of characteristics associated with weight in any of the experimental treatments and morning/lunch-time sessions (LL 220-225).**

16. L 231-235: If the order of the treatments was not randomized, please provide precise information on how it was done, and add to the discussion possible implication of the non-randomized order on your results.

**We have added a sentence to this effect in the revised manuscript (LL. 239-248). Please see also the first paragraph in our response under comment #5 above and also our response to comment #17 below.**

17. L 233-235: The fact that the behavior is repeatable does not rule out the possibility of an interaction with the treatment in many different directions, please acknowledge this in the manuscript.

**We have demonstrated in an earlier study [9] that PCI duration is reproducible. This means that we found no evidence the distribution (and its central value) changed over 1 to 3 days. Therefore, the effects of treatment and the order of treatment are not confounded. This reproducibility was measured on 2mm-deep sand, which is very close to the depth of the Shallow sand treatment in the present study. The analysis we carried out for our response under comment #3 above shows no evidence that the type of substrate interacts with such reproducibility because there is no difference in the results on Paper between individuals treated 1-3 days earlier on Shallow or Deep sand. We have added a point to this effect to the revised manuscript (LL. 239-248).**

18. L 239-240: What was done with the measurements from these individuals?

**We have clarified that the data for these two individuals were used in all analyses (LL. 253-254). They are highlighted as outliers in the caption for Fig. 6 and their paths and movement/immobility patterns are included in S3-S8 Figs.**

19. L 245-249: Please delete from L 245, merge or rephrase.

**We have rephrased this section and hope the meaning is now clear (LL. 260-268).**

20. L 254-256: How were they defined? For instance, have you considered movements of non-locomotory appendage as movement or not? Please clarify.

**The start of a movement period was defined in the same way as the termination of PCI [9]. This included movements of non-locomotory appendages, whether it was a jerk of the abdomen or an opening of the mandibles. Usually such movements were part of a displacement. A movement period was considered to have terminated when there was no movement, as defined above, for more than a couple of seconds. Note that all these manual measurements were corroborated by the image analysis (S6-S8 Figs.) We have clarified this point in the new version of the manuscript (LL. 270-276).**

21. L293-296: Shall it not be possible that the antlions moved and returned to a shorter-range distance in between the initial (after dropping) and final measurements? If that is the case, please discuss this possibility in the manuscript.

**Yes, it is possible, but that is in the nature of diffusive processes. With the analysis of the distance from the position of dropping (in the Petri dish centre) to the position of final measurement, we wanted to test for any difference in diffusion between the antlions on the three substrates. We have presented the individual paths of all antlions on all substrates in Figs. S3-S5.**

22. L 305-306: The use of the word movement withing brackets twice in this sentence (for immobility duration and successive immobility), seems to be somewhat misleading, may you please clarify it?

**Apologies. We have rephrased this sentence and made similar changes to another place in the manuscript where we had used the same clumsy approach to save space (LL. 325-326, 349).**

23. L 325: Which sample size you are mentioning to be large? Please specify.

**We are referring to the sample sizes for the stated models. They are large because the modelled data include between 2 and 16 repeated measures for each antlion on each of three substrates.**

24. L 559: shouldn't the first movement after the PCI be expected to be the fastest? In case they noticed right after falling that they cannot burrow in that surface.

**Not really when the putative predator that dropped the antlion might still be around.**

25. L 638 [628]: The argument that you may have not found the expected differences due to the small sample size can be extended to the risk of incurring in type 1 error, and as such it is desirable to add a paragraph about the limitations of the findings of this study.

**This is actually a Type II error, the probability of failing to reject Ho when it is true. Please see our response and actions under comment #4 above.**

26. L 640: see also Segovia et al (2019). Curr Zool 65: 553-558.

**Thank you. We have added this reference (LL. 690-695).**

27. L 643-645: Very important suggestion!

**Thank you.**

28. Table 2: Maybe the lack of difference in the slope between Shallow and Deep Sand is related to the small sample size of these groups? Shall it not be worthy of a discussion?

**Yes, this is possible. Please see our response and actions under comment #4 above.**
