# Supplementary material for: Seeking safety: Movement dynamics after post-contact immobility
Source: PLoS One. 2024 Aug 22;19(8):e0307370. doi: 10.1371/journal.pone.0307370 (PMC11340899; doi:10.1371/journal.pone.0307370)
Supplement: Attachment — Submitted filename: Response to Reviewers PONE-D-23-29142R3.docx [file pone.0307370.s019.docx]

**PONE-D-23-29142R3**

**Seeking safety: movement dynamics after post-contact immobility**

Additional Editor Comments:

We are almost there! After this new review round the remaining reviewer asked for very minor changes to be made and as soon as the new version is submitted, I beleive the manuscript will be accepted.

**Thank you. We have implemented the two minor changes requested by Reviewer #5. For the benefit of readers, we have now also uploaded the R scripts for the fitted statistical models as requested by Reviewer #5 under point #10 of the previous round.**

Reviewer #5:

1. The authors carefully answered my comments, and added two paragraphs in the discussion section, as well as multiple fragments of information across the text. The information included in the revised version improved the clarity of the manuscript concerning the methods used and its limitations. In my opinion, the manuscript has improved a lot and is now ready for publication. However, I still have two minor comments that I suggest the authors to consider:

**Thank you.**

2. 1- Adding metadata to a dataset improves its readability, as well as the reproducibility of the results. As such I would recommend the authors to look at the link (<https://the-turing-way.netlify.app/reproducible-research/rdm/rdm-metadata#rr-rdm-metadata> ) and consider to add a data dictionary (<https://help.osf.io/article/217-how-to-make-a-data-dictionary> )

**Thank you for this explanation. We have now provided such metadata for S1 Table by uploading a data dictionary entitled “Franks_etal_Data Dictionary_PLoSOne.xlsx”.**

3. 2- For the comment/answer discussed in the last round below:

“23. L 325: Which sample size you are mentioning to be large? Please specify.

We are referring to the sample sizes for the stated models. They are large because the modelled data include between 2 and 16 repeated measures for each antlion on each of three substrates. I would suggest the authors to add this information in the text, because I was surprised to read it at first, as it seemed to me that the authors were mentioning the actual sample size of the experiment. Adding a sentence could help the reader to not be misguided.

**We have now added this information to the text and the respective section now reads:**

**“Although the small p-values mean the null hypothesis that the residuals are consistent with a normal distribution is rejected, this is to be expected to some extent given that the sample sizes are very large (between two and 16 repeated measures for each of between eight and 22 antlions on each of three substrates).” [LL. 345-349]**
